# Longitudinal Multi-Parametric Liquid Biopsy Approach Identifies Unique Features of Circulating Tumor Cell, Extracellular Vesicle, and Cell-Free DNA Characterization for Disease Monitoring in Metastatic Breast Cancer Patients

**DOI:** 10.3390/cells10020212

**Published:** 2021-01-21

**Authors:** Corinna Keup, Vinay Suryaprakash, Markus Storbeck, Oliver Hoffmann, Rainer Kimmig, Sabine Kasimir-Bauer

**Affiliations:** 1 Department of Gynecology and Obstetrics, University Hospital of Essen, 45122 Essen, Germany; Oliver.Hoffmann@uk-essen.de (O.H.); rainer.kimmig@uk-essen.de (R.K.); sabine.kasimir-bauer@uk-essen.de (S.K.-B.); 2 QIAGEN GmbH, 40724 Hilden, Germany; vinay.suryaprakash@qiagen.com (V.S.); Markus.Storbeck@qiagen.com (M.S.)

**Keywords:** multi-parametric, multi-modal, multi-analyte, follow-up, serial sampling, gene expression, molecular signature, mutation, next-generation sequencing, unique molecular indices

## Abstract

Dynamics of mRNA from circulating tumor cells (CTCs), mRNA from extracellular vesicles (EVs), and cell-free DNA (cfDNA) were assessed to examine the relevance of a longitudinal multi-parametric liquid biopsy strategy. Eighteen milliliters of blood was drawn from 27 hormone receptor-positive and human epidermal growth factor receptor 2 (HER2)-negative metastatic breast cancer (MBC) patients at disease progression and at two subsequent radiologic staging time points. CTC mRNA and EV mRNA were analyzed using multi-marker qPCR, and cfDNA was analyzed using targeted next-generation sequencing (NGS). The presence of *ERBB2* or *ERBB3* overexpression signals in CTCs significantly correlated with disease progression (87% specificity, 36% sensitivity, *p*-value = 0.023), and the presence of either *ERBB3* signals in CTCs or EVs or cfDNA variants in *ERBB3* also showed a significant association with progressive MBC. Fluctuations during treatment were detected in the EV fraction with the appearance of hitherto undetected *ERCC1* signals correlating with progressive disease (97% specificity, 18% sensitivity, *p*-value = 0.030). Allele frequency development of *ESR1* and *PIK3CA* variants detected at subsequent staging time points could be used as a predictor for therapy success and, importantly, might help guide therapy decisions. The three analytes, each with their own unique features for disease monitoring, were shown to be complementary, underlining the usefulness of the longitudinal multi-parametric liquid biopsy approach.

## 1. Introduction

Liquid biopsies provide analytes that are powerful for disease monitoring and for the identification of molecular disease features [1,2]. In contrast to spatially and temporally limited tissue biopsies, liquid biopsies can serve as a real-time snapshot to mirror tumoral heterogeneity [3]. Besides the determination of somatic alterations and disrupted pathways, liquid biopsy testing harbors great potential for sensitive detection of minimal residual disease (MRD) or therapy resistance and, consequently, recurrence or disease progression [4].

In breast cancer (BC), the leading cancer in women worldwide [5], an increase in circulating tumor cell (CTC) count [6] and an increase in circulating tumor DNA (ctDNA) concentration have been shown to correlate with disease progression [7,8]. More specifically, in metastatic BC (MBC) patients, a reduction of the apoptotic CTC count by ≥50% (after one completed treatment cycle) correlated with stable disease, while a reduction of apoptotic CTCs ≤ 10% was specific for early disease progression in 74% of the cases [9]. In primary BC, the persistent detection of Cytokeratin-19-positive CTCs during the first 5 years after surgery has been related to an increased risk of late relapse [10]. 

Besides a serial analysis of CTCs, sequencing of cell-free DNA (cfDNA) sampled during the course of treatment has frequently been shown to provide monitoring information. For the detection of MRD in a primary BC setting, mostly patient-specific sequencing assays were used to enhance sensitivity [11]. In MBC, the disappearance of truncating cfDNA *PIK3CA* mutations after 2 weeks of treatment with palbociclib predicted sensitivity to this cyclin dependent kinase (CDK) 4/6 inhibitor and better progression-free survival (PFS) [12]. Furthermore, an increase in the *PIK3CA* variant allele frequency (VAF) in cfDNA has been shown to be associated with acquired resistance to paclitaxel, while an increased representation of cfDNA *RB1* or *MED1* mutations was correlated with acquired resistance to tamoxifen plus trastuzumab [13]. Moreover, the disappearance of *RASSF1A* methylation, detected in cfDNA after adjuvant tamoxifen treatment, indicated response [14]. Persistent *ESR1* methylation in CTCs of advanced hormone-receptor (HR)-positive human epidermal growth factor receptor 2 (HER2)-negative BC patients was related to a lack of response [15]. Occurrence of cfDNA *ESR1* mutations under aromatase inhibitor therapy in HR+ MBC patients correlated with progression [16] and loss of these specific mutations during the course of endocrine treatment was shown to be related to a longer response [17]. On the other hand, in HER2+ MBC patients, a decrease in copy number variations in the *ERBB2* gene on anti-HER2 treatment was associated with a clinical response and has been described as a monitoring marker [18,19]. 

These studies showed numerous single liquid biopsy analytes which are applicable for monitoring purposes. However, the question which of these analytes might be the most suitable one and, more importantly, whether a combination of analytes might increase the sensitivity or specificity of disease progression detection, still remains open. 

Here, we studied the mRNA expression profile of CTCs and extracellular vesicles (EVs), along with the mutational profile of cfDNA, all isolated from only 18 mL blood from HR+ HER2– MBC patients at three time points during the course of treatment. With this longitudinal multi-parametric liquid biopsy approach, we aimed to identify a marker (consisting of one or more parameters) for disease monitoring, compare the compatibility of the different analytes, and investigate the potential additive value of the three analytes. 

## 2. Patients and Methods

### 2.1. Patients

Blood samples from 27 MBC patients were studied. All participants were ≥18 years, had no second malignancies and no severe co-morbidities, but had Eastern Cooperative Oncology Group (ECOG) scores for performance status of 0–2. All kinds of prior treatment of BC were permitted. MBC patients had estrogen receptor (ER) and/or progesterone receptor (PR)-positive primary tumors (summarized as hormone receptor-positive (HR+)) but HER2-negative primary tumors (DAKO score 0–1 or DAKO score 2 with negative in situ hybridization results). Patients with ER-positive and/or PR-positive and HER2-negative metastases were also included if their ER, PR, and HER2 status in the primary tumor was unknown (*n* = 5). All patients showed a progressive MBC at the time of first blood draw/initial time point (TP0). The blood draw was then repeated at the two subsequent staging time points (TP1 and TP2). Consequently, blood specimens at three time points, each characterized by staging via computed tomography (CT) or magnetic resonance imaging (MRI) and evaluated according to the RECIST criteria [20], were obtained and portrayed the disease across a treatment duration of about 9 months (staging every third month). Non-responders were classified as having progressive disease (progress, PD), which was characterized by at least a 20% increase in the sum of the longest diameter of target lesions. Responders showed the following RECIST evaluation: complete response, partial response, or stable disease (SD). 

The results from the first blood draw at TP0 have already been used for a comparison of cfDNA with genomic DNA of CTCs [21], and the results from all three blood draws have already been used for CTC mRNA and EV mRNA comparisons [22].

Patient characteristics are listed in Supplementary Table S1. Written informed consent was obtained from all participants during enrollment and specimens were collected using protocols approved by the Ethics Committee of the University Hospital of Essen (12-5265-BO).

### 2.2. Sample Collection and Liquid Biopsy Analyte Extraction

Two samples of 9 mL EDTA blood were collected and stored for a maximum of 4 h at 4 °C. CTCs were isolated in duplicate from 5 mL of whole blood by positive immunomagnetic selection targeting EpCAM, EGFR, and HER2 (AdnaTest EMT-2/StemCell Select^TM^, QIAGEN) [22]. The CTC-depleted blood remaining after positive immunomagnetic selection [23], as well as the remaining blood (not used for CTC isolation) were centrifuged at 1841× *g* for 8 min and the obtained plasma was frozen at −80 °C. EVs were isolated from 4 mL prefiltered (0.8 µm pore size) plasma by affinity-based binding to a spin column [22,24]. Subsequently, the total RNA was isolated and purified (exoRNeasy Kit, QIAGEN). The mRNA was isolated from the CTC lysates and from the vesicular RNA eluates by Oligo(dT)_25_ beads and was reverse transcribed (AdnaTest EMT-2/StemCell Detect^TM^, QIAGEN) [22]. cfDNA was isolated by affinity-based binding to magnetic beads (QIAamp MinElute ccfDNA Kit, QIAGEN), as previously described [25], using plasma from CTC-depleted blood (≥1 mL, preferentially the maximal available volume; mean: 4.6 mL). cfDNA quantification was performed using the Agilent Chip High Sensitivity DNA assessing the concentration of all fragments with lengths between 100 and 700 bp.

### 2.3. Quantitative PCR

For CTC and EV mRNA profiling, multi-marker RT-qPCR was performed with the AdnaTest TNBC Panel prototype (QIAGEN), already described in detail [22]. Transcript-specific multi-plex pre-amplification was followed by a SYBR green-based qPCR with the StepOnePlus™ (Life Technologies) real-time system analyzing 17 transcripts (namely, *AKT2, ALK, AR, AURKA, BRCA1, EGFR, ERCC1, ERBB2, ERBB3, KIT, KRT5, MET, MTOR, NOTCH1, PARP1, PIK3CA, SRC*, plus *CD45,* and *GAPDH*) in single-plex reactions. Melting curves were obtained to exclude the results of unspecific amplicons. Potential PCR inhibition and contamination were checked by an artificial RNA spike and negative controls. Data evaluation was performed according to previously published protocols [22]. In brief, transcripts not exclusively expressed in CTCs were normalized to the leukocyte-specific transcript *CD45* (also known as *PTPRC*). CTC and EV expression data of the patients were normalized to matched expression data of healthy donor controls and signals were analyzed binarily (overexpression yes/no). In contrast to cellular overexpression being a reason for the overrepresentation of a transcript in CTCs, the reasons for the overrepresentation of a transcript in EVs, also termed here as “overexpression”, may include (1) the overexpression of the transcript in donor cells, (2) selective export of the transcript into EVs, (3) the increased stability of those EVs carrying the transcript, or (4) enhanced release of EVs of the cell population expressing the transcript.

### 2.4. Sequencing

The cfDNA libraries were constructed with a customized QIAseq Targeted DNA Panel Kit (QIAGEN) targeting all exonic regions of 17 genes (namely *AKT1, AR, BRCA1, BRCA2, EGFR, ERBB2, ERBB3, ERCC4, ESR1, KRAS, FGFR1, MUC16, PIK3CA, PIK3R1, PTEN, PTGFR,* and *TGFB1*) as previously described in detail [21]. The preferred input amount for library preparation was in the range of 30–60 ng, but cfDNA samples with a lower input were also included in the library preparation (mean DNA input per sample: 44 ng). Libraries were quantified by qPCR (those with a yield of <4 nM were excluded) and the quality was checked using Agilent Chip High Sensitivity DNA. All pooled libraries were analyzed by paired-end sequencing on an Illumina NextSeq instrument using the NextSeq 500/550 High Output Kit v2.5 with 2 × 150 bp reads.

Bioinformatic analysis of the raw sequencing data was performed on the basis of a pipeline previously described [21]. Sufficient sequencing quality of all samples was guaranteed by the exclusion of cfDNA libraries with fewer than 4 million read fragments, which simultaneously excluded all samples with a unique molecular index (UMI) coverage lower than 400 and samples that only had ≤94% of the target region covered with at least 5% of the mean UMI coverage. The input amounts, library yield, and sequencing quality parameters for each sample are summarized in Supplementary Table S2. For analysis, we used the next-generation sequencing (NGS) Analysis service for QIAseq Targeted DNA Panels available at QIAGEN’s GeneGlobe, which allowed reliable variant calling based on UMI information. Ingenuity Variant Analysis (IVA; QIAGEN) was further used for annotation, scoring, filtering, and interpretation of the resulting variant files. All filter settings are described in detail [21]. All called variants and their corresponding allele frequencies are listed per patient and time point in Supplementary Table S3.

### 2.5. Statistical Analysis

**Figure 1:**
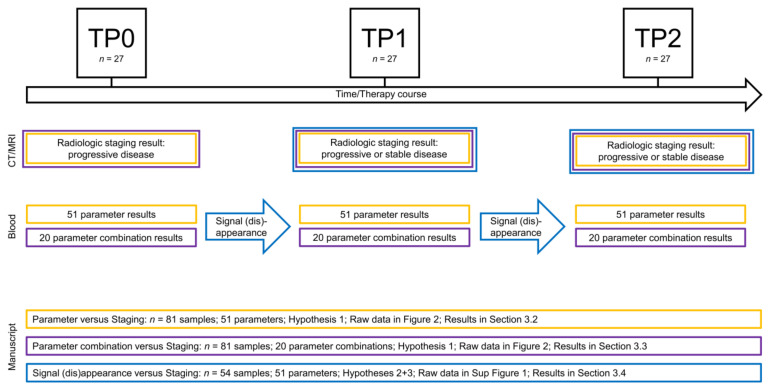
Data evaluation workflow.

The data evaluation workflow is visualized in Figure 1.

To identify a monitoring marker, we first expressed

**Hypothesis** **1.** *that the presence of a parameter correlates with progressive disease proven by radiologic imaging at the same time point (results in Section 3.2).*

To test this hypothesis and the others to follow, we defined true positives (parameter yes, progress yes), true negatives (parameter no, progress no), false positives (parameter yes, progress no), and false negatives (parameter no, progress yes). Sensitivity, specificity, and accuracy were calculated as follows:

$$\mathrm{Sensitivity}=\frac{\sum \mathrm{True}\;\mathrm{positives}}{\sum \mathrm{all}\;\mathrm{samples}\;\mathrm{at}\;\mathrm{disease}\;\mathrm{progression}}\;,$$

$$\mathrm{Specificity}=\;\frac{\sum \mathrm{True}\;\mathrm{negatives}}{\sum \mathrm{all}\;\mathrm{samples}\;\mathrm{not}\;\mathrm{at}\;\mathrm{disease}\;\mathrm{progression}}\;,$$

$$\mathrm{Accuracy}=\;\frac{\sum \mathrm{True}\;\mathrm{positives}+\sum \mathrm{True}\;\mathrm{negatives}}{\sum \mathrm{all}\;\mathrm{samples}}\;.$$

Parameters were sorted according to their sensitivity, specificity, and accuracy as well as according to the sum of their respective sensitivities, specificities, and accuracies.

Additionally, an evaluation of the correlation of a parameter’s presence with the staging results was performed using a two-tailed Fisher’s exact test with significant *p*-values defined as <0.05 (SPSS, version 11.5). To adjust for multiple testing, we performed the Holm–Bonferroni correction with α = 0.05 [26].

Furthermore, we formulated

**Hypothesis** **2.** *that the appearance of a signal not present at the previous time point correlates with progressive disease proven by radiologic imaging (Section 3.4).*

We also hypothesized

**Hypothesis** **3.** *that the signal disappearance from one time point to the next time point is correlated with response. The evaluation of sensitivity, specificity, and accuracy, as well as the two-tailed Fisher’s exact test and its adjustment, were then carried out as described above.*

The stochastic equivalence between the non-binary data of the difference in VAF of *ESR1* and *PIK3CA* variants and staging results was examined using the two-tailed Mann–Whitney U-test (SPSS, version 11.5).

Diagrams were computed with OriginPro version 2019 (OriginLab Corporation) and Microsoft Excel (Microsoft Corporation). 

## 3. Results

### 3.1. Longitudinal Multi-Parametric Liquid Biopsy Approach

The patient cohort consisted of 27 HR+ HER2– MBC patients (Supplementary Table S1). At primary diagnosis, most patients presented with ductal BC and histologic Grade 2 tumors, tumor size T2, and no metastasis. At the initial blood draw (TP0), all patients had metastases and at the time of data evaluation, 6/27 patients were still alive while the others had died. The median follow-up time (first diagnosis of BC to death/last contact) was 136 months (interquartile range: 114).

**Figure 2:**
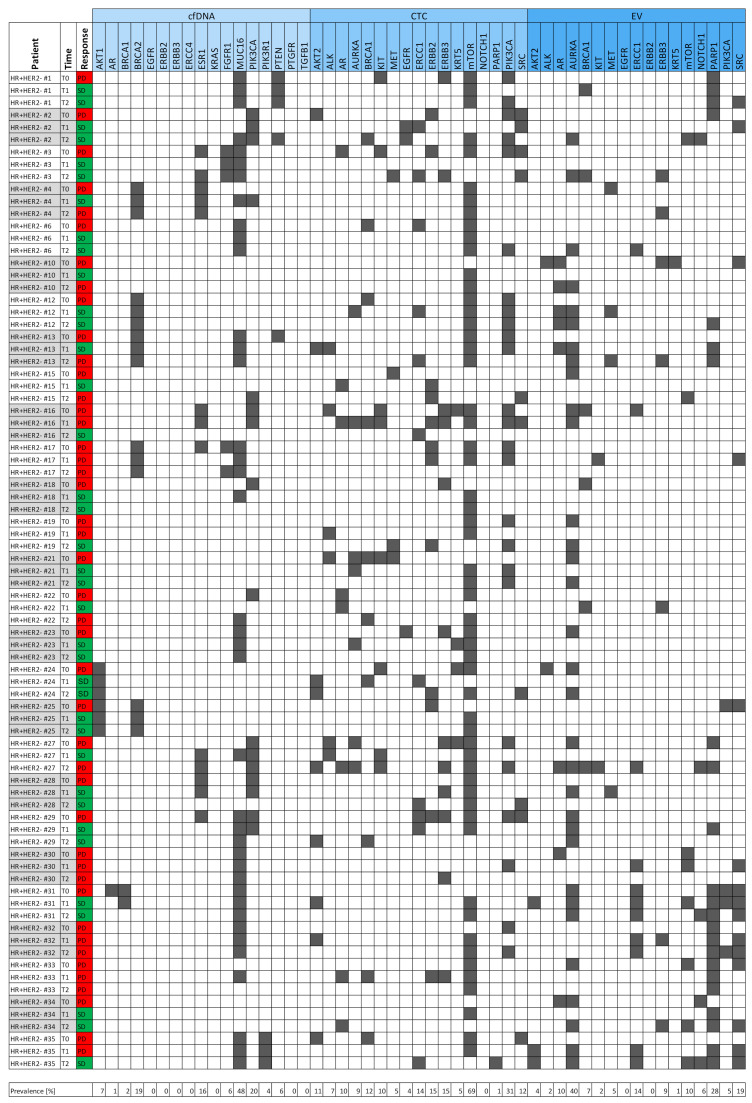
Heatmap of the entire data matrix. Three blood samples from the initial progression time point (TP0) and two subsequent staging time points (TP1 and TP2) of each of the 27 HR+ HER2– metastatic breast cancer (MBC) patients were used to characterize 17 parameters in each of the three liquid biopsy analytes: cell-free DNA (cfDNA, light blue), circulating tumor cell (CTC) mRNA (blue), and extracellular vesicle (EV) mRNA (dark blue). The presence of a variant (cfDNA) or overexpression signal (CTC or EV) is denoted by gray squares. The results of the radiologic imaging performed at the time of blood draw divided the samples into two clinically relevant populations [progressive disease (PD), in red; stable disease (SD), in green].

Patient specimens were only included in this multi-parametric liquid biopsy study if sufficient material existed for matched CTC isolation (10 mL whole blood), EV isolation (4 mL plasma), cfDNA isolation (≥1 mL plasma from CTC-depleted blood), and reliable sequencing result interpretation (>4 nM library yield, >4 million reads fragments per sample). As this is a longitudinal study, patient samples were only included if the abovementioned inclusion criteria were fulfilled for all three blood samples drawn at the three consecutive staging time points (progressive disease TP0 and the two subsequent staging time points, TP1 and TP2). Consequently, the sample cohort consisted of 81 samples (three samples from each of the 27 patients) with three liquid biopsy analytes characterized in each sample. The characterization of each analyte included the results of 17 parameters in each analyte due to a multi-marker qPCR with 17 transcript-specific assays for CTC and EV mRNA profiling and targeted NGS with 17 genes of interest for cfDNA mutational analysis. In summary, the data matrix consisted of 81 samples and 51 parameters (17 parameters in each of the three analytes; Figure 2).

Of the 81 samples, 44 samples were drawn at a time point when progressive disease was proven by radiologic imaging, while the remaining 37 samples were obtained at time points at which the patients were characterized as having a stable disease (including complete response and partial response).

The heatmap visualizes the data matrix (Figure 2) by dividing the 51 parameters into the three analytes, i.e., cfDNA, CTCs, and EVs. *MUC16* variants were the most prevalent variants (48%), while *mTOR* overexpression signals were the most common signals in CTCs (69%). *ERBB2* and *ERBB3* overexpression signals in CTCs were both detected with a prevalence of 15%. Overall, the largest number of signals occurred within the CTC fraction (186 overexpression signals) when compared with 131 overexpression signals in the EV fraction and 105 variants in the cfDNA fraction.

Moreover, we hypothesized that the appearance of variants or signals not detected at the prior time point would be correlated with progressive disease (Hypothesis 2 in Section 2). Supplementary Figure S1 depicts the 54 samples in which we analyzed whether the signals hitherto undetected (at the previous time point) appeared. Signal appearance was just as prevalent in the CTC fraction (70 signals) as in the EV fraction (62 signals), while only 18 cfDNA variants newly occurred. Thus, the mRNA profiles of CTCs and EVs were shown to be similarly dynamic across treatment, but the genomic information obtained from cfDNA remained roughly stable during the 9 months (three time points, staging every third month). In almost a quarter of all patients (24%), EV *AURKA* signals appeared during treatment, thereby representing the most dynamic parameter within the dataset. In contrast, EV *ERCC1* signals appeared in only 9% of all cases, which is slightly more common than the mean signal appearance of all CTC and EV parameters (7%).

### 3.2. Signals as Monitoring Markers

**Figure 3:**
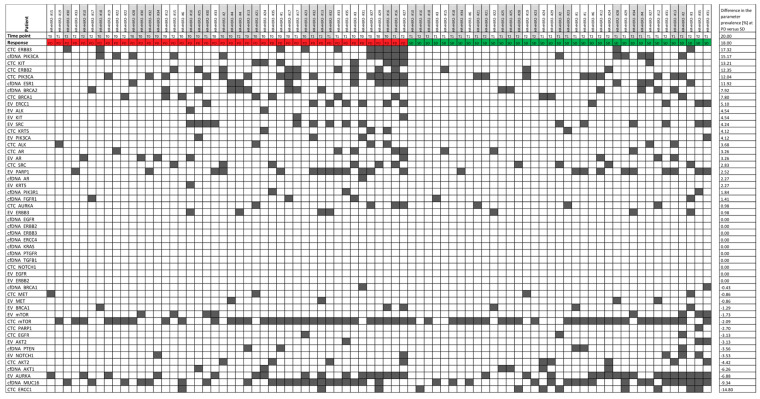
Heatmap of the signal prevalence in samples drawn at a progressive disease time point (PD, red) versus stable disease time point (SD, green). The 81 samples were divided into the population of samples drawn at PD (44 samples) and SD (37 samples). The signal prevalence (in %) of all 51 parameters was calculated in the two populations separately and was then compared by calculating the difference in the prevalence within the two populations.

The parameters were sorted according to the difference in their prevalence at the PD time point versus the SD time point (Figure 3). CTC_*ERBB3*, cfDNA_*PIK3CA*, CTC_*KIT*, CTC_*ERBB2*, CTC_*PIK3CA*, cfDNA_*ESR1*, cfDNA_*BRCA2*, CTC_*BRCA1*, and EV_*ERCC1* were more prevalent in the PD samples when compared with the SD samples. Interestingly, CTC_*ERCC1*, cfDNA_*MUC16*, EV_*AURKA,* and cfDNA_*AKT1* were more common (difference in prevalence > 5%) in SD samples when compared with PD samples. A two-tailed Fisher’s exact test showed that none of the signals was significantly correlated with disease progression. Hypothesis 1 (Section 2) was therefore proven to be false, meaning that the presence of single parameters is not useful for monitoring.

Specificity calculations for each parameter revealed 14 parameters (mostly cfDNA variants and EV signals) with 100% specificity (Supplementary Table S4), implying that these signals were only present in PD samples. The only parameter with a sensitivity of >50% was CTC_*mTOR* with 68% sensitivity (Supplementary Table S4), while mostly CTC signals (CTC_*mTOR* among others), cfDNA_*ESR1,* and cfDNA_*PIK3CA* showed an accuracy of >50%. CTC_*ERBB3* and CTC_*PIK3CA* had the highest accuracies, with 56% and 54%, respectively.

### 3.3. Parameter Combinations as Monitoring Markers

It is worth examining whether a combination of parameters using a disjunction (i.e., a logical OR operation) would result in monitoring markers that are more suitable than single parameters. Not all 1275 pairwise combinations of two parameters were tested, but the parameters with the best sensitivity were combined with those having the best specificity, namely:
EV_*AURKA*ORcfDNA_*ERCC4*EV_*PARP*ORcfDNA_*ERCC4*CTC_*ERBB3*ORcfDNA_*ERBB2*CTC_*ERBB3*ORcfDNA_*ERBB3*CTC_*ERBB3*
OREV_*ERBB2*CTC_*ERBB2*ORcfDNA_*ERBB2*CTC_*ERBB2*ORcfDNA_*ERBB3*CTC_*ERBB2*OREV_*ERBB2*

Additionally, disjunctions of two parameters with the best sensitivity were also tested:
CTC_*mTOR*ORCTC_*PIK3CA*CTC_*PIK3CA*ORcfDNA_*PIK3CA*CTC_*ERBB3*ORCTC_*ERBB2*cfDNA_*ESR1*ORcfDNA_*PIK3CA*

As some of the same genes and transcripts were tested in all three analytes, combinations of these parameters were also tested for their ability to be used as monitoring markers:
cfDNA_*AKT1*
ORCTC_*AKT2*OREV_*AKT2*cfDNA_*AR*
ORCTC_*AR*OREV_*AR*cfDNA_*BRCA1*
ORCTC_*BRCA1*OREV_*BRCA1*cfDNA_*EGFR*
ORCTC_*EGFR*OREV_*EGFR*cfDNA_*ERCC4*
ORCTC_*ERCC1*OREV_*ERCC1*cfDNA_*ERBB2*
ORCTC_*ERBB2*OREV_*ERBB2*cfDNA_*ERBB3*
ORCTC_*ERBB3*OREV_*ERBB3*cfDNA_*PIK3CA*
ORCTC_*PIK3CA*OREV_*PIK3CA*

None of the parameter combinations above showed a specificity of 100%, but CTC_*mTOR* OR CTC_*PIK3CA* and cfDNA_*PIK3CA* OR CTC_*PIK3CA* OR EV_*PIK3CA* showed a sensitivity of >50% (Supplementary Table S4). CTC_*ERBB3*, CTC_*PIK3CA,* and CTC_*ERBB2* were the most frequently occurring parameters among the combinations with the best accuracy (Supplementary Table S4). Importantly, two significant correlations with disease progression were identified by the two-tailed Fisher’s exact test, namely: CTC_*ERBB3* OR CTC_*ERBB2* (*p*-value = 0.023), and cfDNA_*ERBB3* OR CTC_*ERBB3* OR EV_*ERBB3* (*p*-value = 0.032). Some parameter combinations were thus shown to validate Hypothesis 1.

### 3.4. Signal Appearance as Monitoring Marker

Here, we examined whether signal dynamics during treatment were more informative as monitoring markers than signals at a given time point. First, we analyzed the data to test Hypothesis 2: whether the appearance of a signal hitherto undetected at the previous time point was correlated with progressive disease (Supplementary Figure S1).

Signal appearance showed 100% specificity in 20/51 parameters—mostly cfDNA variants (Supplementary Table S4). While none of these parameters showed a sensitivity of > 50%, all parameters performed with an accuracy of >50%. Seven out of 51 parameters showed an accuracy of >70%, with EV_*ERCC1*_appearance being one of them. This is salient, because the appearance of EV_*ERCC1* significantly correlated with disease progression (*p*-value = 0.03 using the two-tailed Fisher’s exact test) and this was significant even after Holm–Bonferroni adjustment for multiple testing, thereby validating Hypothesis 2.

Furthermore, it is noteworthy that the appearance of the combinations CTC_*ERBB3* OR CTC_*ERBB2* and cfDNA_*ERBB3* OR CTC_*ERBB3* OR EV_*ERBB3* was not significantly correlated with disease progression and the signal disappearance of any parameter from the first time point to the subsequent time point was not related to stable disease. As a result, Hypothesis 3 was rejected.

### 3.5. The Best Monitoring Markers 

In order to evaluate all parameters in the three categories “parameters”, “parameter combinations”, and “signal appearance” together, the parameters were sorted in descending order based on the sum of their respective sensitivity, specificity, and accuracy values. Following this, the 15 best potential monitoring markers were selected (Table 1). Most of the top 15 parameters originated from the category “signal appearance” indicating that observing the dynamics across treatment by evaluation of serial liquid biopsies is more informative for disease monitoring than the detection of variants or overexpression signals at a single time point. 

**Figure 4:**
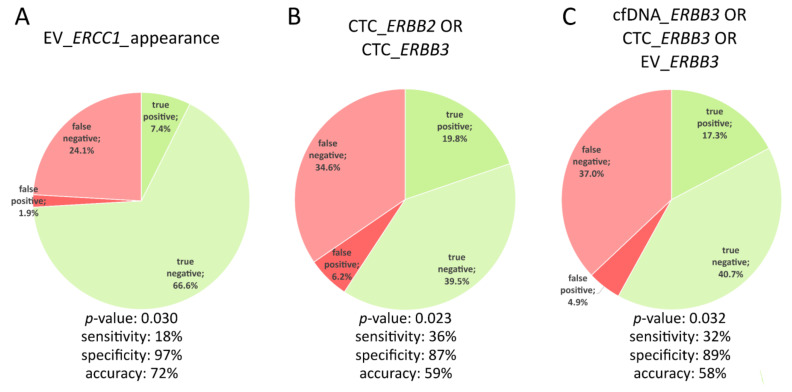
Characteristics of the three monitoring markers identified. A significant correlation (*p*-value < 0.05) between the signal appearance (**A**) or parameter combination (**B + C**) and disease progression was shown using the two-tailed Fisher’s exact test. The accuracy of the three monitoring markers decreases from left to right.

As mentioned in Section 3.3 and Section 3.4, EV_*ERCC1* appearance, the disjunction of CTC_*ERBB2* and CTC_*ERBB3*, and the disjunction of the three *ERBB3* analytes were shown to significantly correlate with disease progression. These three parameters were also listed among the top 15 potential monitoring markers with the greatest sum of sensitivity, specificity, and accuracy values (Table 1). The difference between the accuracy of the two parameter combinations is not significant (with accuracies of 58% and 59%, respectively) owing to almost similar fractions of false positives, false negatives, true positives, and true negatives (Figure 4). In comparison, the appearance of EV_*ERCC1* was characterized by a higher accuracy of 72%. The detection of newly occurring *ERCC1* overexpression signals in EVs from one time point to the next had a specificity of 97% (only 1.9% false positives) but the rate of true positives was just 7.4% (sensitivity of 18%) (Figure 4).

### 3.6. Allele Frequency Development of ESR1 and PIK3CA Variants

In accordance with the finding in Section 3.5 that the molecular dynamics detected in longitudinal liquid biopsies were, in general, very informative and taking into account the clinical relevance of *ESR1* variants and *PIK3CA* variants for MBC treatment management, it is worth evaluating whether the appearance of *ESR1* or *PIK3CA* cfDNA variants is a reliable indicator for disease monitoring. In the entire cohort, the accuracy for detecting disease progression was 70% and 66% for *PIK3CA* and *ESR1* variant appearances, respectively (Supplementary Table S4), and cfDNA_*PIK3CA*_appearance ranged among the top 15 parameters in Table 1.

**Figure 5:**
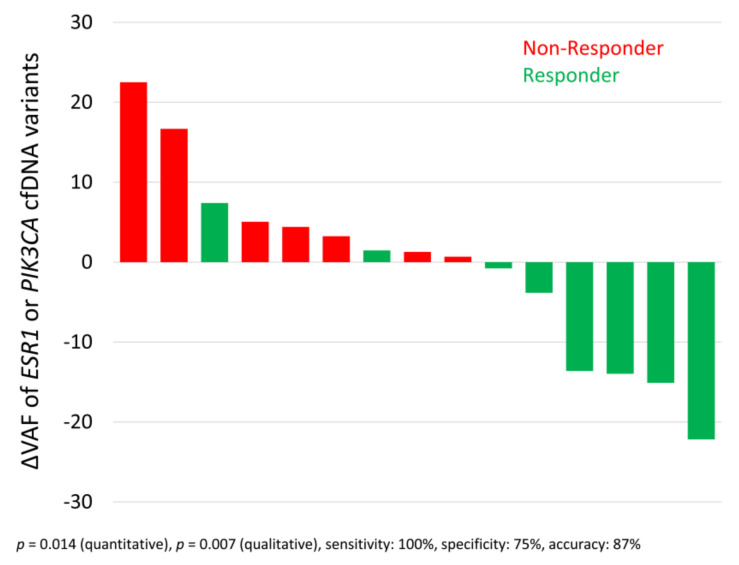
Waterfall plot mapping the cfDNA variant allele frequency (VAF) development of *ESR1* or *PIK3CA* variants and therapy response. Difference in variant allele frequencies (ΔVAF) of *ESR1* variants or *PIK3CA* variants between two consecutive staging time points correlated with therapy response (at the second time point evaluated by radiologic imaging and according to RECIST). Only the 15 cases with a detectable VAF at one time point and the next time point were evaluated. The two-tailed Mann–Whitney U-test and the two-tailed Fisher’s exact test were used to evaluate the ∆VAFs thus obtained.

If the VAF is considered as a non-binary parameter, it is possible to compute differences in VAF between two time points (ΔVAF). For samples with variants detected at two consecutive time points (*n* = 15), the difference between the VAFs at the two time points was computed. The waterfall plot depicts a significant correlation of *ESR1* OR *PIK3CA* allele frequency development with disease progression, here called non-response (Figure 5; *p*-value = 0.014 using the two-tailed Mann–Whitney U-test). Qualitative analysis of the increase or decrease in VAF also revealed a significant correlation in these 15 samples with disease progression (*p*-value = 0.007 using the two-tailed Fisher’s exact test), with an accuracy of 87%. It is noteworthy that neither the standalone evaluation with respect to *ESR1* nor the standalone evaluation with respect to *PIK3CA* VAF reached significance.

### 3.7. Six Index Patients

**Figure 6:**
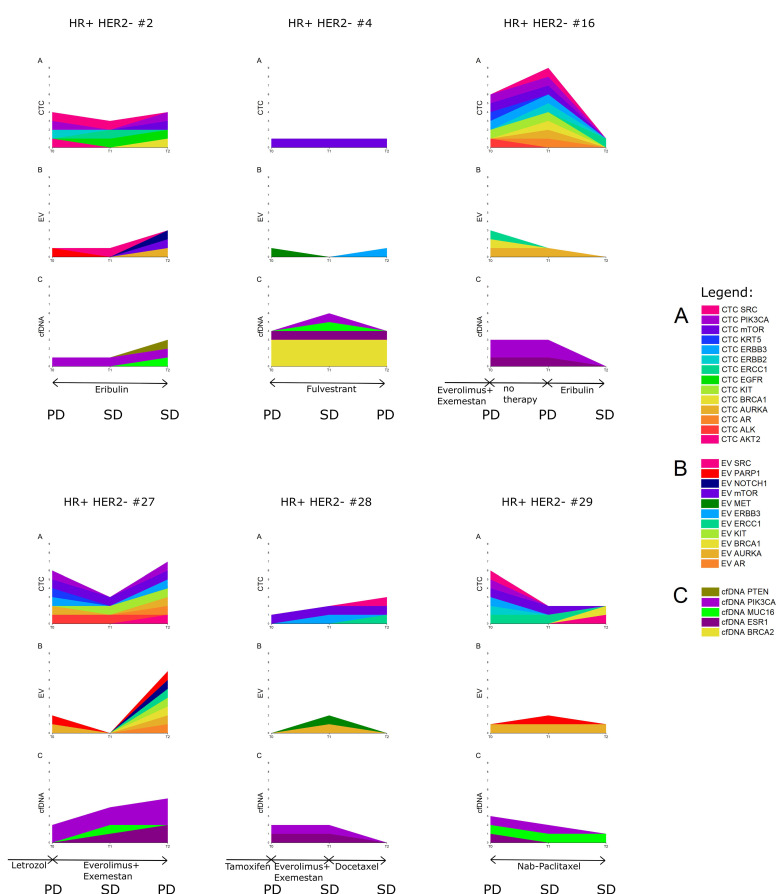
Development of CTC and EV overexpression signals and cfDNA variants in six index patients at three consecutive staging time points. The staging result (by radiologic imaging according to RECIST guidelines) at the time point at which blood was drawn is indicated as PD (progressive disease) or SD (stable disease). (**A**) CTC mRNA signals, (**B**) EV mRNA signals, and (**C**) cfDNA variants. Within each analyte, each color indicates a variant in another gene or overexpression signal of another transcript, while data of the same gene or transcript in different analytes are indicated with the same color.

To understand the value of a longitudinal multi-parametric liquid biopsy strategy in greater detail, six index patients were chosen (Figure 6). All six index patients were characterized by *ESR1* and *PIK3CA* cfDNA variants detected at more than one time point. In the individual patients depicted, it was observed that signals from only a single analyte were particularly prominent at all three given points in time (Supplementary Figure S2). The multi-parametric liquid biopsy approach might, thus, cover a range of inter-patient heterogeneity.

The diversity of CTC signals is illustrated by the different overexpression signals in different colors (Figure 6A, especially in HR+ HER2– #16). In contrast, within the EV and cfDNA fractions, a low number of different signals was detected (with the exception of HR+ HER2– #27 at T2). Especially in the latter patient, large fluctuations in EV signals during treatment were observed. Patients HR+ HER2– #27 and #29 exemplified the appearance of signals or variants at disease progression, whereas the appearance of CTC signals at T2 in patients HR+ HER2– #2 and #28 did not support the hypothesis that disease progression is characterized by signal appearance. In general, the three analytes showed a similar development in signal prevalence at the three time points in patients HR+ HER2– #16 and #29. Interestingly, however, the pattern of signal or variant presence and appearance in the three analytes in patients HR+ HER2– #4, #16, and #28 was dissimilar and indicated the additive value of using multiple analytes. 

More specifically, the prevalence of some of the CTC signals and their matched EVs (e.g., *BRCA1* signals) were observed to be inversely proportional during treatment in patient HR+ HER2– #16, whereas a similar reduction in the prevalence of cfDNA_*PIK3CA* and CTC_*PIK3CA* signals occurred after successful chemotherapy in patients HR+ HER2– #16 and #29. An observation concerning CTC_*ERCC1* signals and chemotherapy: A switch to chemotherapy induced CTC_*ERCC1* signals in one case (HR+ HER2– #28). In three other cases, CTC_*ERCC1* signals persisted despite the patients responding to the administered chemotherapy—proven by radiologic imaging (in patients HR+ HER2– #2, #16, and #29).

From the observations above, although concrete inferences cannot be drawn about an individual analyte being the most conducive monitoring marker, one can recognize the benefits of a longitudinal multi-parametric liquid biopsy strategy in being able to capture tumoral heterogeneity and tumoral evolution.

### 3.8. The Course of cfDNA PIK3CA and ESR1 Variants in Three Index Patients

**Figure 7:**
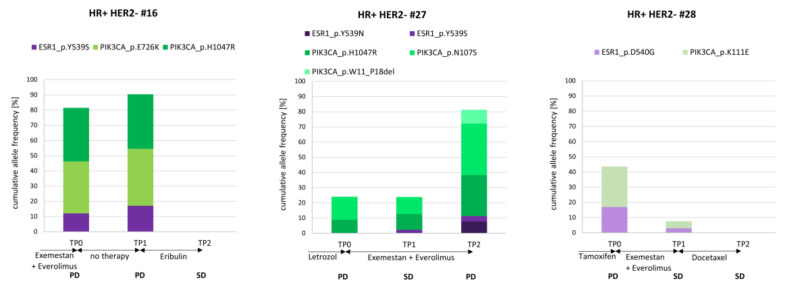
cfDNA variants across three consecutive staging time points. The cumulative allele frequency of all detected cfDNA variants at each of the three time points was exemplified in three patients. Therapy regimens and staging results (SD: stable disease; PD: progressive disease) are indicated. *PIK3CA* (green) and *ESR1* (purple) variants were called in these patients, mostly with varying allele frequencies across the observation period.

To examine the clinically relevant cfDNA_*ESR1* and cfDNA_*PIK3CA* signals more closely, three index patients with variants in these two genes (both detected at two time points at least) were selected (Figure 7). The different variants in the *PIK3CA* gene were color-coded in different shades of green and the different *ESR1* variants with shades of purple. 

Two and one out of three index patients (Figure 7) were polyclonal for *PIK3CA* and *ESR1*, respectively. In patients HR+ HER2– #16 and #27, two *PIK3CA* variants (one variant being *PIK3CA* H1047R) were observed at the progression time point TP0 after endocrine treatment. In the latter patient (HR+ HER2– #27), one new cfDNA variant in the *PIK3CA* gene was detected after the second unsuccessful endocrine treatment line with exemestan plus everolimus, while the two *PIK3CA* variants detected previously demonstrated an increase in VAF. After 3 months with no treatment, the same variants were detected, with increased VAF in patient HR+ HER2– #16. In contrast, after successful chemotherapy, neither *PIK3CA* variants nor *ESR1* variants could be detected in two cases (HR+ HER2– #16 and #28).

Interestingly, *ESR1* Y539S was detected in both cases with disease progression under aromatase inhibitor therapy (HR+ HER2– # 16 and #27). Importantly, in one case (HR+ HER2– #27), the appearance of this *ESR1* variant was documented 3 months before the radiologic imaging determined the disease to be progressive.

## 4. Discussion

Within the last decade, liquid biopsy has become a frequently studied tool with a high potential for disease management [27,28,29], and has gained acceptance for prognosis and treatment decision-making in MBC [30,31]. Since one of the major advantages of liquid biopsies is the possibility for longitudinal assessment by repeated minimally invasive sampling, single liquid biopsies have already been studied as potential monitoring markers in MBC [9,12,13,14,15,17,32]. Here, we conducted a longitudinal multi-parametric liquid biopsy study, including the characterization of CTC mRNA, EV mRNA, and cfDNA—all isolated from a minimized blood volume. By applying this strategy to samples from 27 HR+ HER2– MBC patients at three time points during the course of treatment, we identified markers for disease monitoring, revealed the unique features of the different analytes for monitoring purposes, and, consequently, deliberated on the additive value of using three analytes.

The cohort was composed only of samples from patients whose blood was drawn at the initial progression time point and the two subsequent staging time points thereafter, and samples for which the isolation and reliable analysis of all three liquid biopsy analytes was achieved. These stringent criteria resulted in a dataset containing 51 analyzed parameters and 81 samples, of which half were collected at a stable disease time point, while the other half of the samples were drawn at disease progression, thereby enabling a balanced evaluation of all parameters with regard to disease monitoring. This study is based on the assumption that the visual staging via CT or MRI and evaluation according to RECIST guidelines accurately mirror tumor growth. This assumption, however, prevents us from evaluating the extent to which liquid biopsy results might be more sensitive than radiologic imaging. 

CTC_*ERBB3* as well as CTC_*ERBB2* overexpression signals were prevalent in 15% of all samples. Furthermore, both parameters were more prevalent in the PD samples when compared with SD samples. Among all the parameters considered, CTC_*ERBB3* signals performed the best, with an accuracy of 56%. When combined with CTC_*ERBB2*, CTC_*ERBB3* signals offered a sensitivity of 36%, a specificity of 86%, and an accuracy of 59% in disease monitoring and were significantly correlated with disease progression. The importance of *ERBB3* for MBC patient stratification also became apparent by the significant correlation of the disjunction of the three *ERBB3* analytes (cfDNA_*ERBB3* OR CTC_*ERBB3* OR EV_*ERBB3*) with progression (sensitivity: 32%, specificity: 89%, accuracy: 58%). 

The sequential nature of this study enabled the observation of signal appearances from one time point to the next across 3 months of treatment. The appearance of EV_*ERCC1* signals not present at the previous time point was a significant indicator for disease progression with a specificity of 97% and an accuracy of 72%. However, the sensitivity was only 18%, which stems from a rather low signal prevalence of just 9%. The strength of most “signal appearance” parameters was the increased specificity, but this was dampened by low sensitivity. Along these lines, specific *ERCC1* polymorphisms have been shown to associate with increased BC susceptibility [33], and high ERCC1 protein expression in the primary tissue has been shown to associate with poor outcomes in metastatic triple negative BC patients treated with platinum-based chemotherapy [34]. 

Among the top 15 monitoring parameters (based on the sum of their sensitivity, specificity, and accuracy), most parameters originated from the category “signal appearance”. We therefore conclude that the dynamics during treatment are more informative for disease monitoring than the detection of variants or overexpression signals at a single time point. However, the appearance of the signal combinations identified as monitoring markers, namely CTC_*ERBB3* OR CTC_*ERBB2* and cfDNA_*ERBB3* OR CTC_*ERBB3* OR EV_*ERBB3,* were not significantly correlated with disease progression. Despite many studies showing an effect of variant clearance under therapy [12,17], in this work, we could not prove that signal disappearance from one time point to the next was related to stable disease.

In general, the appearance of new signals was as frequent in the CTC fraction as in the EV fraction. However, the most dynamic parameter originated from the EV fraction, with EV_*AURKA* occurring newly in 24% of all cases. Since most signals were detected in the CTC fraction, the most accurate monitoring parameters also originated from it and a great diversity of CTC signals existed at a given time point (as demonstrated in the index patients). A unique feature of the cfDNA fraction was neither the diversity and high prevalence of signals (as in the CTC fraction) nor the dynamics across treatment (as demonstrated in the EV fraction), but the actionability of detected variants. The success of a liquid biopsy-guided therapy by assessing *PIK3CA, ESR1*, and *AKT1* mutations in the CTCs of four MBCs from successive blood draws has already been demonstrated in a proof-of-concept study [35]. While Yanagita et al. preferred cfDNA monitoring over CTC monitoring [36], here, the advantages and disadvantages of cfDNA and CTCs for disease monitoring were shown to be balanced.

Here, we would like to discuss the value of rigorous exclusion criteria for cfDNA variant detection. The expected low VAF of tumor-derived variants necessitated a great diversity of DNA fragments as library input and thus, cfDNA had to be isolated from a large plasma volume [37,38]. A mean of 4.6 mL plasma from CTC-depleted blood was used for cfDNA isolation, and after sequencing, only the samples with more than 4 million read fragments were included for the analysis. The latter criterion indirectly led to the exclusion of samples with <400 UMI coverage. The high sequencing depth, the use of UMIs, and the other aforementioned criteria resulted in a good limit of detection of 1.3% across all target regions, meaning that variants with VAFs of ≥1.3% were called with a probability of 90%. However, for a detailed analysis of specific *PIK3CA* and *ESR1* variants in the index patients, we refrained from including non-detected variants for VAF difference calculation, since we could not rule out false negative results (despite the good limit of detection mentioned above).

In accordance with the benefit of alpelisib for *PIK3CA* mutant MBC patients [30] and the endocrine resistance caused by *ESR1* variants [39,40,41,42,43], we observed a difference in the VAFs of detectable variants in both genes and showed them to be significantly correlated with disease response when the two were jointly analyzed. Within the entire cohort, cfDNA_*ESR1* and cfDNA_*PIK3CA* showed an accuracy of >50% for disease monitoring but, due to their low prevalence, a significant correlation with disease progression could not be found. In six patients, the detection of *PIK3CA* variants was described at the time point of progression under endocrine treatment and *ESR1* variants occurred in relation to unsuccessful aromatase inhibitor therapy. In one case, we even detected a newly occurring *ESR1* variant 3 months before radiologic imaging detected the disease to be progressive. The acquired dominance of an *ESR1* variant has already been demonstrated for serial monitoring in one exemplary case by Schiavon et al. in 2015 [43]. *PIK3CA* variants seem to relate to tumor burden in the index patients, marked by an increase in VAF at PD, and a dramatic decrease in VAF after successful chemotherapy. In line with these results, it has already been shown that *PIK3CA* variants reflect responses to therapies more accurately than carcinoembryonic antigen (CEA) or carcinoma antigen 15-3 (CA15-3) [8] and that changes in *PIK3CA* ctDNA levels correlated with treatment response [37,44].

Interestingly, only signals from a single analyte were particularly prominent at any given point in time for a specific patient, indicating that the multi-parametric liquid biopsy approach could prove advantageous in reflecting inter-patient heterogeneity. The multi-parametric approach also provides additive value when compared with single liquid biopsy strategies due to dissimilar fluctuations observed in the three analytes over time.

It is important to mention that the multi-parametric liquid biopsy approach requires the isolation and molecular analysis of three analytes, thereby resulting in high costs. Reimbursement of liquid biopsy testing was shown to save costs by preventing the administration of ineffective drugs and avoiding the need for multiple tissue biopsies [45]. This leads us to surmise that multi-parametric liquid biopsy testing could become more widely accepted by healthcare systems in the future and thereby make reimbursements easier. However, to the best of our knowledge, full financial coverage of a multi-parametric approach is currently unavailable.

The study’s major limitation is the lack of a germline control. Analysis of variants within the buffy coat DNA could have identified some cfDNA variants which are actually germline variants. Furthermore, buffy coat DNA could have captured somatic variants originating from clonal hematopoiesis of indeterminate potential (which increases with age) and not from the solid MBC [46].

Furthermore, it is of note that the sensitivity of the identified monitoring markers is low and thus utilizing them in clinical practice might be difficult. The advantage of these markers, however, is the high specificity. Consequently, detection of the monitoring markers would indicate disease progression with high probability, while the absence of the markers might not suffice to describe a SD. 

Another crucial aspect of our work is the multiple testing. The same dataset was used to test significant correlations of disease response with the 51 parameters and with some parameter combinations. Additionally, a reduced dataset was used to assess the correlation of progression and the fluctuations in the presence of the 51 parameters. This multiple testing increased the Type I errors and was consequently adjusted using the Holm–Bonferroni correction [26]. However, this adjustment led to an increase in Type II errors, translating into the fact that significant correlations with progression were missed (false negative results). It is to be noted that in this study, we did not want to prove that all tested parameters correlated with disease progression, which is the ultimate goal of Bonferroni corrections [47]. Moreover, we also did not compare the *p*-values, but just used the two-tailed Fisher’s exact test to examine a potentially significant correlation between the response and a parameter’s presence. These arguments led us to the conviction that adjustment for multiple testing is not mandatory in this study, which is in accordance with Perneger [47]. Nonetheless, here, we state that CTC_*ERBB3* OR CTC_*ERBB2* and cfDNA_*ERBB3* OR CTC_*ERBB3* OR EV_*ERBB3* did not significantly correlate with disease progression after adjustment for multiple testing, while the appearance of EV_*ERCC1* signals was significantly correlated with progression even after Holm–Bonferroni adjustment.

## 5. Conclusions

In conclusion, the characterization of each of the three analytes showed unique features for monitoring purposes. While CTC mRNA profiles were diverse and commonly found, mirroring the spatial tumoral heterogeneity, EV signals fluctuated greatly across treatment, thereby mirroring the temporal heterogeneity. The VAF development of the less frequent but actionable variants *ESR1* and *PIK3CA* in cfDNA, and a combination of the *ERBB3* and *ERBB2* signals in CTCs, or the appearance of *ERCC1* signals in EVs were found to be suitable for monitoring. Along these lines, none of the analytes is favored for disease monitoring, but the virtues of a longitudinal multi-parametric liquid biopsy strategy in being able to deconvolute tumoral heterogeneity and tumoral evolution are presented.

## Figures and Tables

**Table 1:** The top 15 parameters with the greatest sum of sensitivity, specificity, and accuracy values. The parameters in bold were shown to significantly correlate with disease progression.

Parameter	Sensitivity	Specificity	Accuracy	Sensitivity + Specificity + Accuracy
**EV_ERCC1_appearance**	0.1765	0.9730	0.7222	1.8717
EV_KIT_appearance	0.1176	1.0000	0.7222	1.8399
CTC_AR_appearance	0.1765	0.9459	0.7037	1.8261
CTC_ERBB3_appearance	0.1765	0.9459	0.7037	1.8261
**CTC_ERBB3ORCTC_ERBB2**	0.3636	0.8649	0.5926	1.8211
cfDNA_PIK3CA_appearance	0.1176	0.9730	0.7037	1.7943
**cfDNA_ERRB3ORCTC_ERBB3OREV_ERBB3**	0.3182	0.8919	0.5802	1.7903
CTC_BRCA1_appearance	0.1765	0.9189	0.6852	1.7806
EV_ERBB3_appearance	0.1765	0.9189	0.6852	1.7806
EV_SRC_appearance	0.1765	0.9189	0.6852	1.7806
cfDNA_FGFR1_appearance	0.0588	1.0000	0.7037	1.7625
EV_PIK3CA_appearance	0.0588	1.0000	0.7037	1.7625
CTC_AURKA_appearance	0.1176	0.9459	0.6852	1.7488
EV_AR_appearance	0.1176	0.9459	0.6852	1.7488
CTC_ERBB3	0.2273	0.9459	0.5556	1.7288

## Data Availability

The sequencing data presented in this study are available in Supplementary Tables S2 and S3.

## Supplementary Materials

The following are available online at https://www.mdpi.com/2073-4409/10/2/212/s1. Supplementary Figure S1: Heatmap of signal appearance. The appearance of cfDNA variants (light blue) and CTC (blue) or EV (dark blue) overexpression signals was calculated between time points TP0 to TP1 and between TP1 and TP2, and then correlated with the staging result (PD, *n* = 17, red; SD, *n* = 37, green) at TP1 or TP2, respectively. The signal appearance is denoted by gray squares. Supplementary Figure S2: Radar plot with the number of different signals or variants in the three analytes. Each radar plot depicts a given patient and the changes in the three analytes observed at various points in time are depicted by planes of different colors. Supplementary Table S1: Patient characteristics. Supplementary Table S2: Sequencing quality parameters for cfDNA sequencing. Supplementary Table S3: cfDNA variants and corresponding variant allele frequencies (VAFs) called after the described stringent variant filtering listed for all patients (*n* = 27) at all three time points. Supplementary Table S4: Summary of the parameters with the best sensitivity, specificity, and accuracy and a significant correlation with disease progression. For the categories “parameters”, “parameter combinations”, and “signal appearance”, the parameters with specificity = 100%, or sensitivity > 50%, or accuracy > 50%, or a significant correlation with disease progression based on the two-tailed Fisher’s exact test (*p*–value < 0.05) are listed.

## Abbreviations

BC	Breast cancer
CDK 4/6	Cyclin dependent kinase 4/6
CEA	Carcinoembryonic antigen
CA 15-3	Carcinoma antigen 15-3
cfDNA	Cell-free DNA
CT	Computed tomography
CTCs	Circulating tumor cells
ctDNA	Circulating tumor DNA
ΔVAF	Difference in variant allele frequencies
ECOG	Eastern Cooperative Oncology Group
ER	Estrogen receptor
EVs	Extracellular vesicles
HR	Hormone-receptor
HER2	Human epidermal growth factor receptor 2
IVA	Ingenuity variant analysis
NGS	Next-generation sequencing
MRI	Magnetic resonance imaging
MBC	Metastatic breast cancer
MRD	Minimal residual disease
PD	Progressive disease progress
PFS	Progression-free survival
PR	Progesterone receptor
SD	Stable disease
VAF	Variant allele frequency
**List of genes**	
*AKT1*	AKT serine/threonine kinase 1
*AKT2*	AKT serine/threonine kinase 2
*AR*	Androgen receptor
*AURKA*	Aurora kinase A
*BRCA1*	BRCA1 DNA repair associated
*BRCA2*	BRCA2 DNA repair associated
*EGFR*	Epidermal growth factor receptor
*EpCAM*	Epithelial cell adhesion molecule
*ERCC1*	ERCC excision repair 1, endonuclease non-catalytic subunit
*ERCC4*	ERCC excision repair 4, endonuclease non-catalytic subunit
*ERBB2*	Erb-b2 receptor tyrosine kinase 2 coding for the HER2 protein
*ERBB3*	Erb-b2 receptor tyrosine kinase 3
*ESR1*	Estrogen receptor 1
*FGFR1*	Fibroblast growth factor receptor 1
*GAPDH*	Glyceraldehyde-3-phosphate dehydrogenase
*KIT*	KIT proto-oncogene receptor tyrosine kinase
*KRT5*	Keratin 5
*KRAS*	KRAS proto-oncogene, GTPase
*MET*	MET proto-oncogene, receptor tyrosine kinase
*mTOR*	Mechanistic target of rapamycin
*MUC16*	Mucin 16, cell-surface associated
*NOTCH1*	Notch 1
*PARP1*	Poly(ADP-ribose) polymerase 1
*PIK3CA*	Phosphatidylinositol-4,5-bisphosphate 3-kinase catalytic subunit alpha
*PIK3R1*	Phosphoinositide-3-kinase regulatory subunit 1
*PTEN*	Phosphatase and tensin homolog
*PTGFR*	Prostaglandin F receptor
*PTPRC*	Protein tyrosine phosphatase, receptor type, C (also known as CD45)
*SOX17*	SRY-box transcription factor 17
*SRC*	SRC proto-oncogene, non-receptor tyrosine kinase
*TGFB1*	Transforming growth factor beta 1
