# Supplementary figures and images for: Longitudinal Multi-Parametric Liquid Biopsy Approach Identifies Unique Features of Circulating Tumor Cell, Extracellular Vesicle, and Cell-Free DNA Characterization for Disease Monitoring in Metastatic Breast Cancer Patients
Source: Cells. 2021 Jan 21;10(2):212. doi: 10.3390/cells10020212 (PMC7912374; doi:10.3390/cells10020212)

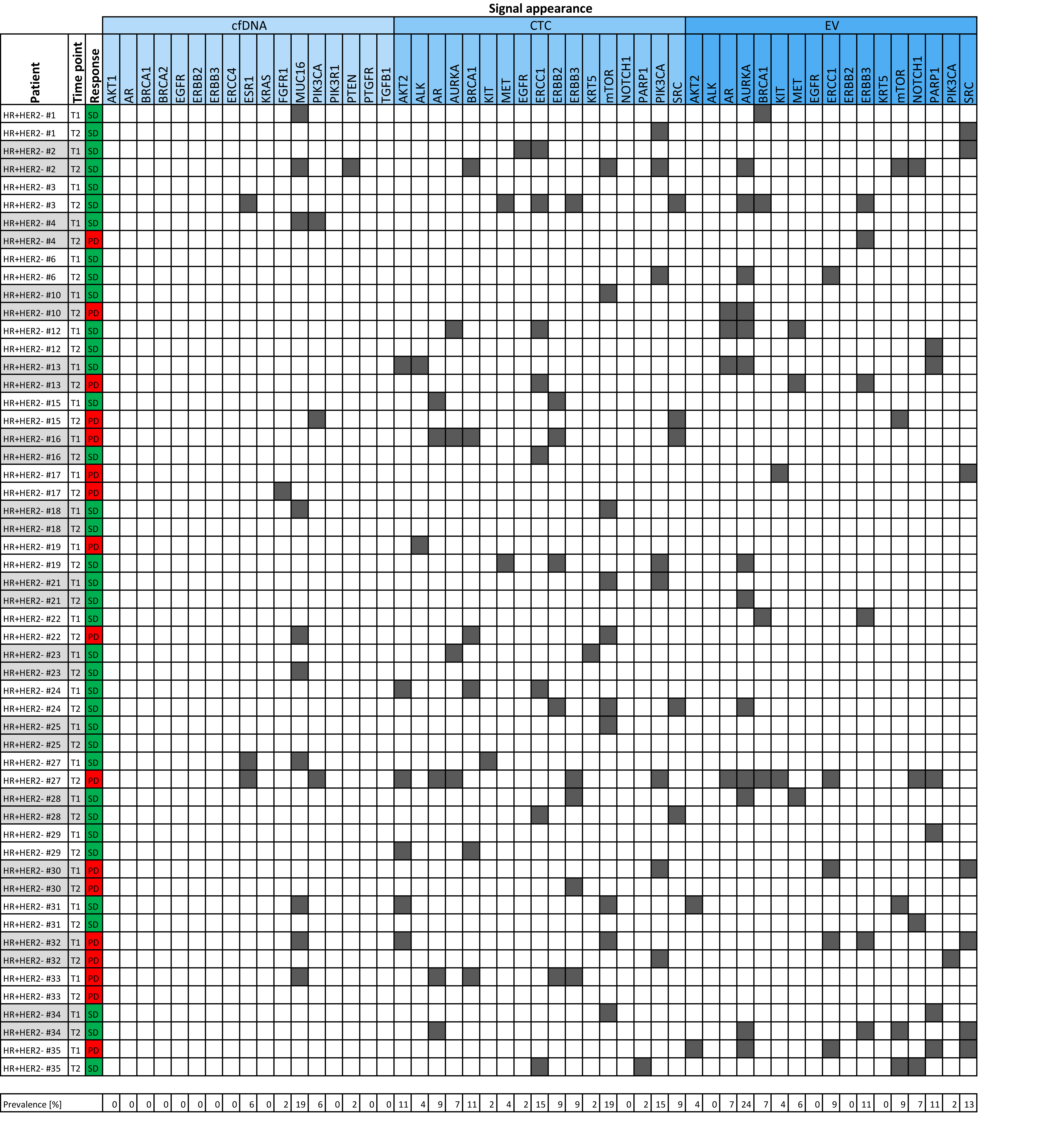

Supplement: Supplementary file 1 [file cells-10-00212-s001.zip › Supplements/Sup Fig 1.tif]

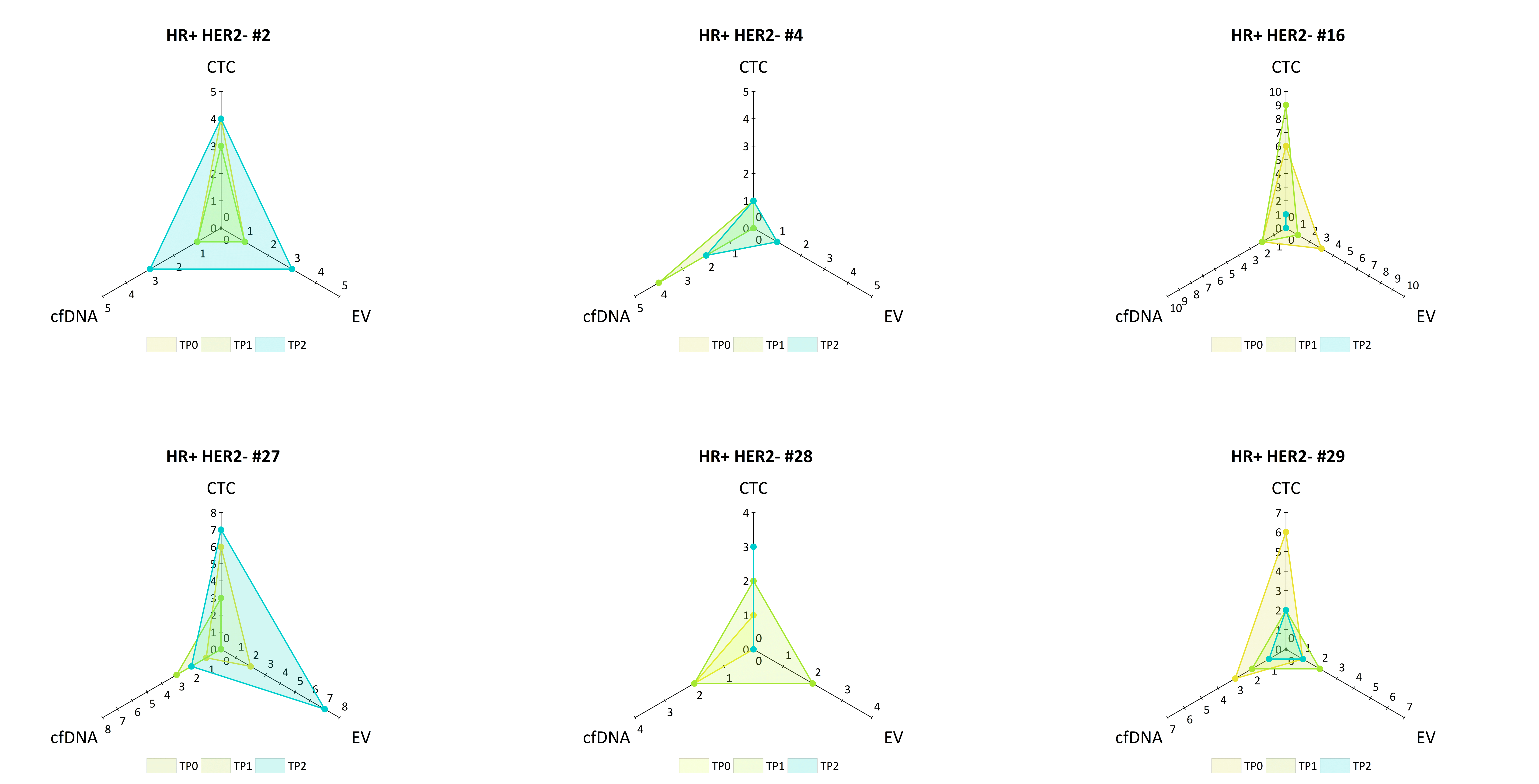

Supplement: Supplementary file 1 [file cells-10-00212-s001.zip › Supplements/Sup Fig 2.png]
